# Medical clowns improve sleep and shorten hospitalization duration in hospitalized children

**DOI:** 10.1038/s41598-024-52943-2

**Published:** 2024-01-29

**Authors:** Maya Shimshi-Barash, Ido Orlin, Tali Jacob, Gali Kushnir, Lara Rawashdeh, Etay Rothem Nachmias, Noam Meiri, Giora Pillar

**Affiliations:** Department of Pediatrics and Pediatric Sleep Medicine, Carmel Medical Center, Technion Faculty of Medicine, 7 Michasl St., 3436212 Haifa, Israel

**Keywords:** Paediatrics, Sleep

## Abstract

Intervention by medical clowns was proven to have a positive effect in reducing stress and anxiety, increasing cooperation and improving the child's experience prior to a medical procedure and during the various stages of hospitalization. Sleep has long been known to be essential for recovery from injury and sickness, improving immune functions, and there is an emerging understanding of the restorative role quality sleep has on health and diseases. Hospitalized children are more exposed to sleep disorders and sleep deprivation due to the hospitalized environment, anxiety, and illness. Different behavioral interventions to promote sleep were previously studied in hospitalized children, some showing potential benefits. In this study, we sought to examine the ability of medical clowns to positively impact the child's sleep during hospitalization. The study is an observational matching (case–control) interventional study which took place at the department of pediatrics in Carmel Medical Center. Forty-two hospitalized children ages 2–17 were included in two equal groups of intervention or control. Children in the control group were recruited based on a method of matching the chief complaint plus the medical diagnosis and age of the children in the intervention group in a 1:1 matching. The children's sleep parameters were objectively evaluated for two consecutive nights using an Actigraph device and subjectively by parent's questionnaire. Additional factors such as hospital length of stay and demographics were also monitored. The study group had an encounter with a medical clown (15–30 min) before bedtime on either the first or the second night, and the control group was not exposed to a medical clown at all. We then compared the data from both groups using unpaired t-tests. Hospitalized children exposed to a medical clown prior to bedtime (n = 21) and children not exposed to a medical clown (n = 21) were comparable in age and clinical characteristics. The study group had a significantly delayed wake-up time compared to the control group (06:59 ± 46 min vs. 07:26 ± 42 min, p < 0.05) (mean difference of 27 min). Night's duration (from bedtime to wake-up) was significantly longer in the study versus the control group (570 ± 76 vs. 500 ± 66.1 min, p < 0.05), a total mean increase of 70 min, and sleep efficiency were significantly increased (92.3 ± 4.6% vs. 87.9 ± 8.7%, p < 0.05). Within the clown group, when comparing nights with and without exposure to a medical clown, total sleep time was prolonged by a mean of 54 min on the night of the intervention (518 ± 74 min vs. 464 ± 59 min, p < 0.01), and the total wake time during the night were reduced (52 ± 27 min vs. 77 ± 61 min, P < 0.05), mean difference of 25 min), mainly by reduction of wake period after sleep onset (WASO) (42 ± 25 min vs. 66 ± 58 min, p < 0.05), mean difference of 24 min). Regarding general medical outcomes, hospital stay was significantly shorter in the clown group vs. control (104 ± 42 h vs. 128 ± 42 h, p < 0.05), a mean reduction of 23 h—nearly an entire day. An encounter with a medical clown before bedtime in hospitalized children positively affects sleep parameters, which may be of great importance for healing in general. The clown intervention was also shown to shorten the hospital stay. Larger scale studies are warranted to establish these findings.

## Introduction

Sleep has long been known to be essential for recovery from injury and sickness, and there is an emerging understanding of the restorative role of sleep in health and disease. Unfortunately, the hospital environment is often poorly accommodating for uninterrupted sleep. Pain, anxiety, medication effects, medical interventions, and the acute illness contribute to decreased quality and quantity of sleep in hospitalized patients. As a result, issues related to sleep and sleep disorders are important to address when considering all the factors influencing the child's wellbeing during inpatient care^1,2^.

Adult patients regularly report poor sleep quality during hospitalization across several inpatient settings^3,4^. Patients have cited poor sleep quality as one of the main stressors during hospital admissions. The implications of hospitalization on sleep quality in the pediatric population may be even more severe due to the anxiety and the negative effect of drastic changes in their immediate environment^5^. In a study examining the sleep quality of hospitalized, non-ICU, pediatric patients, younger children reported later bedtimes, later wake-up times, more nighttime awakenings, and shorter total sleep time while hospitalized. Adolescents had later wake-up times, more nighttime awakenings, and shorter total sleep time during hospitalization. Parents reported later bedtimes, later awake times, and more nighttime awakenings when rooming in^6^. A recent scoping review that included 20 different studies assessing sleep duration in hospitalized children found that in the great majority of studies (16/20), mean sleep duration was shorter than age-appropriate recommended sleep duration (as recommended by the American National Sleep Foundations)^7^. Reported causes of sleep disturbances were related to modifiable external factors (e.g., nursing care activities and noise from equipment and other patients) and internal factors experienced by the child, such as pain and worries.

The consequences of acute and chronic sleep deprivation are increasingly recognized. Data on the impact of poor sleep quality and loss of sleep during hospitalization is more limited. As detailed above, sleep deprivation and disruption are reported as very common in hospitalized patients; thus, its effects on physical and mental well-being, as studied in different settings, would seem to apply to the inpatient setting as well and requires further investigation. Sleep deprivation may affect the function of the immune and inflammation response^8,9^. A study on glucose control in both diabetics and nondiabetics found an inverse relation between the duration of sleep in the hospital and glucose levels^10^. Acute sleep deprivation is well recognized to increase anxiety and reduce the pain threshold^11,12^. Sleep deprivation also affects the respiratory system, cognitive function, and more^13–17^. Ultimately, sleep deprivation during hospitalization may impact recovery from acute illness^18^.

Many aspects of the acute care hospital environment can adversely influence sleep, especially noise, patient care interruptions, and alterations in the light–dark cycle^19^. These factors only add to the inherent challenges of sleeping in an unfamiliar environment and internal factors disrupting sleep in the hospitalized child, namely anxiety and pain.

Various non-pharmacologic interventions have been proposed to help enhance sleep quality and duration in hospitalized patients^20^, such as improving the sleep environment, reducing light and noise interference, etc. Some studies showed the benefit of relaxation techniques and their positive effect on sleep in hospitalized patients. A recent Cochrane review concluded the findings of ten studies on non-pharmacological sleep promotion interventions in hospitalized children^21^. Single studies showed that objective sleep parameters and subjective sleep measures improved following behavioral interventions, including massage, touch therapy, and bedtime stories.

As a treatment modality designed to reduce stress and anxiety in hospitalized children, medical clowns may have great potential for improving inpatient sleep quality in the pediatric population^22^.

Clowning is a kind of ancient art that produces an interaction between people, play, and above all, laughter. The activity of medical clowns in hospital wards has been gaining more and more momentum in recent decades, yet their presence in medical facilities is certainly not a new phenomenon. We can find in the literature evidence of clowns' activity back in the early twentieth century. Later on, in the 1970th, Dr. Patch Adams used clowning skills as part of the hospital treatment of admitted children in Virginia and later became world renowned for setting new concepts and idealizing medical clowning and making it a standard in pediatric medical care. In the 1980s, the first training program for medical clowns was founded, and similar programs were later established worldwide^23^.

The ability of the medical clown to influence the course of healing and recovery in hospitalized children in different aspects has been investigated extensively for the past two decades. Studies demonstrated the positive effects of medical clowns on the pre-procedural emotional state prior to the medical interventions or during induction of anesthesia, on general well-being during hospitalization, compliance with physical examinations, adherence to therapy, and even on treatment outcomes^22–30^. A few studies showed that interacting with a medical clown during anesthesia or before a painful medical procedure reduced anxiety and improved psychological adjustment^24–26^. One randomized controlled trial showed that patients who had a medical clown intervention demonstrated a lower pre-operative anxiety index upon and after surgery, required less induction time for anesthesia, spent overall less time in the operative room, needed less time to recover from surgery and to be discharged home^27^. Pain scores improved during painful interventions in the pediatric emergency care setting, and crying periods were shorter when a medical clown was present^28,29^. Furthermore, the presence of the medical clowns increased cooperation and helped the medical staff conduct the physical examination and medical procedures^24,30–33^. On the biochemical level of evidence, it was shown in a number of studies conducted on hospitalized children and adolescents that salivary cortisol levels were lower after a session with a medical clown compared with the pre-intervention state^34,35^.

Not only children are positively influenced by the presence of a medical clown. Studies showed that the interaction with the medical clown reduced distress in parents and health professionals^36^. Different studies emphasized the positive effects of medical clowns on the child's emotional state and physical wellbeing^37^. Concerning medical outcomes, some studies have demonstrated that medical clowns may have a positive influence on the success rate of medical therapy, such as in the case of success rates of IVF therapy shown to be improved in a study performed in Israel^38^. Incorporating medical clowns into the routine care of hospitalized children can dramatically improve their experience and distract them from the stressful hospital environment; this can improve sleep quality and benefit the child's healing even further.

Reviewing current literature, we found a lack of investigation of the influence of medical clowns on sleep in hospitalized children. This study aimed to evaluate the ability of medical clowns to improve sleep quality in hospitalized children. We hypothesized that interacting with a medical clown can enhance sleep quality in hospitalized children. This, in turn, may promote health restoration and decrease hospitalization length in children.

## Methods

The Carmel IRB committee approved the study for human subject studies, and a parent had to give informed written consent before recruitment. All methods were performed in accordance with the relevant guidelines and regulations. The study was a single-center prospective observational matched trial conducted between July 2019 to January 2022 in the Department of Pediatrics, The Lady Davis Carmel Medical Center in Haifa, Israel. The research participants were recruited based on order of admission. The population relevant for recruitment to the study was pediatric patients with a predicted hospital stay of at least two consecutive nights, under the inclusion criteria (see below). Patients in the control group were recruited based on matching statistical technique, in a 1:1 case–control matching based on age and clinical condition.

Inclusion criteria comprised children ages 2–18 years who required hospitalization, predicted for at least two nights—these included children with appendicitis, urinary tract infection, etc. Exclusion criteria consisted of children younger than two years of age, children 18 years and older, children whose caregivers could not be explained the terms of participation and give informed consent, children with a known sleep disorder, children who received medication that may affect sleep patterns, children with chronic medical conditions (e.g., cystic fibrosis, Down syndrome etc.), children with a known fear of clowns (based on previous experience), and children with expected one-day hospitalization (such as minimal head injury, croup, etc.). Conditions in which the participants were dropped from the study: in the case that the child was discharged home after a single night or transferred to another medical facility, and in the case that the child took off the actigraph device before completing two nights in the study.

Enrolled participants were recruited into two groups and were followed for two consecutive days. In the study group, children were managed according to the usual management protocols for their medical condition and received the trial's studied intervention—a private session engaging with a medical clown adjacent to bedtime. The medical clown used various techniques to relax the patient (e.g., music, singing, guided imagination). Each encounter was held for approximately 15–30 min.

Children in the control group were recruited based on a matching method to be matched by medical condition and age, and if possible by gender (i.e. once a five-year-old child with appendicitis was recruited, the next five-year-old child who was admitted to our ER was attempted to participate as a control). Participants in the control group were managed according to the usual management protocols for their medical condition, precisely like the control study group, apart from medical clowning. Participants in both groups wore an Actigraph device during their hospitalization, which objectively evaluated their sleep parameters. In both groups, the child's primary caregiver, who accompanied them during the hospital stay, was required to answer a three-part questionnaire about:

1. The child's regular sleeping habits at home,

2. the child's sleeping characteristics during two consecutive days in his current hospital admission,

3. Questions for the clown group only inquiring the caregiver's subjective estimation of the clown's influence on the child's sleep.

In compiling and analyzing the data, first data offloaded from the Actigraph device was compared to the subjective parents' questionnaires. Secondly, data on each child in the study group was compared to the corresponding child in the control group.

In order to reduce confounding covariance, we applied a controlled matched trial in which results on each day of admission were compared to the correlating day in the control group.

Statistical analysis was performed using an independent unpaired two tailed t-test, as appropriate for the continuous variables. Differences in sleep and hospitalization parameters between the two hospitalization days in each group separately were analyzed using paired t-test. P < 0.05 was considered statistically significant.

### Ethical approval

The study has been approved by the Carmel Medical Center IRB (approval number 0197-18-CMC) caregivers of all participants had signed the informed consent prior to participation.

## Results

Overall, 57 children were recruited. Fifteen children were dropped from the study for not completing the protocol for two nights (for different reasons, such as taking the actigraph device off or being discharged home before completing the required observation period of two nights). Eventually, 42 children were included: the clown group (study group) (n = 21) and the control group—children not exposed to a medical clown (n = 21). Recruited children in the study group were matched according to age and clinical characteristics with children in the control group. The mean age was 10.8 ± 4.4 years in the clown group (range 2–17 years) and 10.9 ± 4.4 years in the control group (same range). In both groups, 47% (n = 10) of subjects were female, and 53% (n = 11) were males. In the clown group, 47% (n = 10) were exposed to a medical clown on their first night, and 53% (n = 11) were exposed to the clown's intervention on their second night. 28% (n = 12) had at least one previous hospitalization in both groups. The medical diagnoses were as follows in the two groups: Acute appendicitis (n = 7), abdominal pain (n = 4), Immune Thrombocytopenic Purpura (n = 2), arthritis (n = 2), other (n = 6, including pyelonephritis, dental abscess, pneumonia, parotitis, facial nerve palsy, cellulitis). These data are presented in Table 1.

**Table 1:** Various diagnoses, age and gender of the participants.

Diagnosis	Research (clown)			Control (no clown)		
	n	Age	Males (n)	n	Age	Males (n)
Appendicitis	7	11.6 ± 3.8	3	7	11.8 ± 4.8	3
Abd. pain	4	11.8 ± 7.0	2	4	12.2 ± 6.6	1
ITP	2	8.6 ± 2.1	1	2	9.1 ± 1.6	1
Arthritis	2	12.9 ± 2.9	1	2	11.6 ± 0.9	1
Other^a^	6	8.7 ± 4.4	4	6	9.8 ± 5.9	5
All	21	10.8 ± 4.4	11	21	10.9 ± 4.4	11

^a^"Other" consists of pyelonephritis (n = 1), dental abscess (n = 1), pneumonia (n = 1), parotitis (n = 1), facial nerve palsy (n = 1), cellulitis (n = 1).

Subjective sleep habits were similar between the groups. The average estimated subjective total sleep time was 9.22 h in the clown group versus 9.24 h in the control group (p = 0.48), and sleep latency was 28.7 min in the clown group versus 20.5 min in the control group (p = 0.3).

The study group had a significantly delayed wake-up time compared to the control group (objectively by actigraphy 07:26 ± 42 min vs. 06:59 ± 46 min vs., p < 0.05) (difference of 27 min); subjectively by questionnaire 07:14 ± 48 min vs 06:51 ± 40 min, p = 0.13 (difference of 23 min). Time in bed was significantly longer in the study group versus the control group (by actigraphy 570 ± 76 vs. 500 ± 66.1 min, p < 0.05), a total increase of 70 min. Subjectively by parents' estimation total sleep time was in the clown group 464 ± 113 min vs 414 ± 120 min in the control group, a total increase of 50 min p = 0.09). The total wake time during the night was shorter by 2 min in the clown group (52 ± 27 vs. 54 ± 23 in the study group, p < 0.01). The mean sleep period time was longer in the study group, 518 ± 74 min, compared with 446 ± 66 in the control group, with a difference of 72 min between the means of the two groups. Still, the difference did not reach statistical significance (p > 0.1). Sleep efficiency was greater by 1.3 percent in the clown group, 92.31% ± 4.6% vs. 91.01% ± 4.7% in the control group. Although it did reach statistical significance level, it is clinically less important as both groups slept surprisingly well despite being ill and hospitalized. The mean wake period after sleep onset (WASO) was also shorter by 2 min in the clown group but not statistically significant (42 ± 26 vs. 44 ± 22, p > 0.1). The mean number of awakenings during the night was lower in the clown group compared to the control group {15 ± 6.6 vs. 13 ± 5.6) but not statistically significant (p > 0.1).

**Figure 1:**
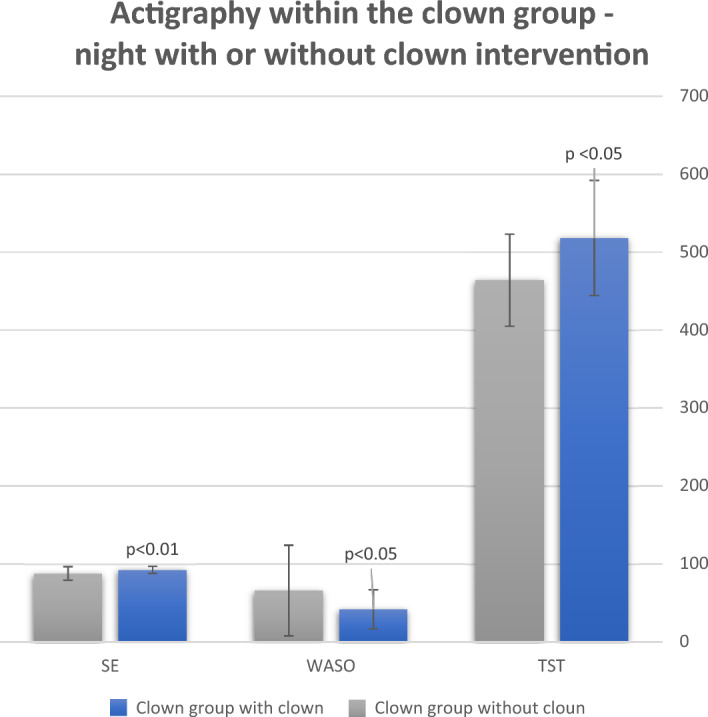
Actigraph results within the clown group—comparing nights with or without clown intervention. *TST* total sleep time (min), *WASO* wake after sleep onset (min), *SE* sleep efficiency (%). As can be seen, on the night with clown intervention all sleep parameters improved.

Within the clown group, when comparing for each child the two nights, with and without the exposure to a medical clown before bedtime—total actual sleep time was prolonged by 54 min on the night after clown exposure (518 ± 74 min vs. 464 ± 59 min, p < 0.01) (Fig. 1). Furthermore, total wake time during the night was reduced (52 ± 27 min vs. 77 ± 61, p < 0.05), mainly by reduction of wake period after sleep onset (WASO) (42 ± 25 min vs. 66 ± 58, p < 0.05, a mean difference of 24 min). Sleep efficiency on the night after clown intervention was greater by 4.3%, compared to the night with no intervention (92.3 ± 4.6% vs. 87.9 ± 8.7%, p < 0.05) (Fig. 1).

Looking at general characteristics of the child's admission other than sleep parameters, there was a significant difference in length of hospital stay: the hospitalization was shorter in the clown group vs. control (104 ± 42h vs. 128 ± 42h, p < 0.05), a difference of 23h, nearly a full day, between groups' means.

## Discussion

In this study, we sought to examine whether an interaction with a medical clown can improve sleep in hospitalized children. Our results show that a 15–30 min session with a medical clown before bedtime positively affected sleep compared to control, including later wake-up time, reduced total wake time during the night, and increased sleep efficiency. When examining the effect of the clown intervention compared to the baseline night without intervention within the study group—we again see a significant improvement in sleep parameters, including total sleep time, WASO, and sleep efficiency. Furthermore, hospitalization length was significantly shorter when the child was exposed to a medical clown before bedtime.

These findings are in concert with previous studies regarding the benefits of medical clowns for improved pediatric patient care, such as the study of Kocherov et al. 2016. Their study examined the interaction with a medical clown on children in a similar age group, 2–16 years old, going through a meatotomy procedure in the operating room. Anxiety level was evaluated using a standardized anxiety scale instrument. They found that children in the study group spent overall less time in the operating room (p < 0.0001) and required less time to recover from the surgery and to be discharged (p = 0.0172)^27^. This study had the advantage of homogeneity (all patents came for meatotomy, but sleep parameters were not assessed.

The findings of improved sleep parameters following the intervention in our study are consistent with previous studies reporting similar results for other relaxing behavioral interventions, including massage and touch therapy^39,40^. The pre-bedtime clown intervention in our research has shown to be even more effective than other behavioral interventions tested in previous studies, including parent-recorded bedtime stories and multicomponent relaxation intervention^33,41,42^. Clowns' activity has been integrated into the medical field in facilities worldwide for several decades. Their activity and influence in various aspects have been investigated. It was found that they have a positive effect at different points in the medical treatment process of hospitalized children as measured by objective parameters of treatment success, as well as on subjective feelings and experiences of the child and parents^23^. However, their effect on sleep has not yet been examined. Therefore, the findings of the study are innovative and further emphasize the positive impact of medical clowns on patients and the paramount importance of their integration into the medical system.

The relationship between the medical clown practice and anxiety level was well established in previous studies. A recent meta-analysis conducted by Kasem Ali Sliman et al. published in 2023 analyzed 18 studies (total of 912 children) indicating significant reduction of procedural anxiety when procedures were performed with a medical clown compared to controls. This meta-analysis concluded that medical clowns have substantial positive and beneficial effects on reducing stress and anxiety in children and their families in various circumstances in pediatrics^43^.

In studies exploring factors influencing sleep, it was well-proven that anxiety harms sleep^44^. Therefore, we believe that the positive effect of medical clowns on the child's sleep, as shown in the study group, is due to their ability to distract the child, mediate and lighten the situation of being hospitalized. Hospitalization can be very stressful for children due to changes in their natural environment, close encounters with strangers, pain and concern due to illness, and medical interventions. The opposite is also true; anxiety level is also influenced by sleep itself, poor sleep quality increases anxiety levels, and it is known that a poor sleeping environment and sleep deprivation during hospitalization could be a tremendous stressor for patients^4–9,18,20,45,46^. Therefore, improving sleep quality and length of sleep during hospitalization would most probably decrease the general anxiety level during hospitalization.

Evaluating sleep in children during hospitalization can pose a certain challenge. Using the entire set up of sleep laboratory polysomnography was not practical. Therefore, we had to use a more user-friendly yet well-validated device such as the Actigraph device. The actigraph device, mainly based on an accelerometer, could only obtain some sleep parameters since our study occurred in a regular pediatric department. Thus, we do not have data regarding sleep stages or potential apnea. Of note, none of the participants in this study demonstrated a fear of clowns, which indeed has been reported to be relatively rare^45^.

The most surprising result in the current study was the dramatic shortening of hospitalization duration in the study group. Mechanistically we assume it can be attributed to better sleep and reduced stress, although other potential mechanisms may also play a role. In terms of potential bias, it should be emphasized that the study team was different than the clinical staff on wards, and the decision when to discharge each child was made by the clinical team and were unrelated to the study. Furthermore, the discharging staff was the regular morning staff and not the physicians on evening/night shifts. Thus, they were unaware of clown intervention or not (unless they actively asked). Therefore, we believe this result is genuine and not due to bias. Many previous studies, including from our group, have demonstrated the effect of better and longer sleep on children, although not necessarily acutely on hospitalization duration^5,17,46,47^. Thus, this potential mechanism remains to be studied in future research.

### Study limitations

Our study has several limitations. First, the sample size is moderate. Due to our matching method and the conduction of the study during the COVID epidemic (when the number of pediatric emergency department visits and hospital admission was significantly reduced), we sometimes had to wait several months to find age and diagnosis matched control, which increased the study duration and limited recruitment^48^. Furthermore, six of the participants were studied prior to the COVID regulations, while the remaining 36 were studied during the COVID pandemic, when clowns wore red-nose plus facemask, which added some inconsistencies. Yet, we believe 42 participants are sufficient to draw at least principal conclusions. Second, the practice of the medical clown is not a standardized interaction method that can be quantified, but rather an approach based on intuitive, flexible communication according to his personality, clowning skills, and type of interaction formed with the child. We tried to minimize this limitation by restricting the study to only two clowns, yet their action is not standardized, and surely differed between small children to adolescents. Future studies should examine the effect of the medical clown with multiple exposers and the long-term effect on sleep and during hospitalizations. Third, the objective assessment of sleep was performed based on actigraphy and not PSG. Thus, we do not have data regarding sleep architecture or stages. Finally, the study was not formally blinded. One cannot build a placebo comparison for a medical clown. We have tried to minimize this effect by blindly analyzing the actigraphy data and by separating the clinical staff from the research staff. Yet, some bias may occur due to the open (un-blinded) fashion of the study. Future studies of greater strength are needed to assess the effects of medical clowns on sleep and associated medical outcomes and hospitalization length, as a positive relation is anticipated due to the critical role of sleep in health and recovery.

## Conclusion

Despite the limitations mentioned above, we believe our study is novel and indicative of the positive effects of medical clowning on hospitalized children. An encounter with a medical clown before bedtime in hospitalized children improves sleep and shortens hospital stays, most probably by reducing anxiety. Nonetheless, larger-scale studies are warranted to support the positive effect of medical clowns on sleep and associated outcomes.

## Data Availability

The datasets used and/or analyzed during the current study available from the corresponding author on reasonable request.

## References

[1] Besedovsky L, Lange T, Born J (2012). Sleep and immune function. Pflugers Arch..

[2] Park MJ (2014). Noise in hospital rooms and sleep disturbance in hospitalized medical patients. Environ. Health Toxicol..

[3] Bano M (2014). The influence of environmental factors on sleep quality in hospitalized medical patients. Front. Neurol..

[4] Hoey LM, Fulbrook P, Douglas JA (2014). Sleep assessment of hospitalised patients: A literature review. Int. J. Nurs. Stud..

[5] Skipper JK, Leonard RC (1968). Children, stress, and hospitalization: A field experiment. J. Health Soc. Behav..

[6] Meltzer LJ, Davis KF, Mindell JA (2012). Patient and parent sleep in a children’s hospital. Pediatr. Nurs..

[7] Hybschmann J (2021). Sleep in hospitalized children and adolescents: A scoping review. Sleep Med. Rev..

[8] Irwin M (1996). Partial night sleep deprivation reduces natural killer and cellular immune responses in humans. FASEB J..

[9] Spiegel K (2002). Effect of sleep deprivation on response to immunizaton. JAMA.

[10] DePietro RH (2017). Association between inpatient sleep loss and hyperglycemia of hospitalization. Diabetes Care.

[11] Pires GN, Bezerra AG, Tufik S, Andersen ML (2016). Effects of acute sleep deprivation on state anxiety levels: A systematic review and meta-analysis. Sleep Med..

[12] Roehrs T, Hyde M, Blaisdell B, Greenwald M, Roth T (2006). Sleep loss and REM sleep loss are hyperalgesic. Sleep.

[13] Goel N, Rao H, Durmer JS, Dinges DF (2009). Neurocognitive consequences of sleep deprivation. Semin. Neurol..

[14] Golan N, Shahar E, Ravid S, Pillar G (2004). Sleep disorders and daytime sleepiness in children with attention-deficit/hyperactive disorder. Sleep.

[15] White DP, Douglas NJ, Pickett CK, Zwillich CW, Weil JV (1983). Sleep deprivation and the control of ventilation. Am. Rev. Respir. Dis..

[16] Ravid S, Afek I, Suraiya S, Shahar E, Pillar G (2009). Sleep disturbances are associated with reduced school achievements in first-grade pupils. Dev. Neuropsychol..

[17] Ravid S, Afek I, Suraiya S, Shahar E, Pillar G (2009). Kindergarten children’s failure to qualify for first grade could result from sleep disturbances. J. Child Neurol..

[18] Hillman DR (2021). Sleep loss in the hospitalized patient and its influence on recovery from illness and operation. Anesth. Analg..

[19] Hu R-F (2015). Non-pharmacological interventions for sleep promotion in the intensive care unit. Cochrane Database Syst. Rev..

[20] Tamrat R, Huynh-Le M-P, Goyal M (2014). Non-pharmacologic interventions to improve the sleep of hospitalized patients: A systematic review. J. Gen. Intern. Med..

[21] Kudchadkar SR (2022). Non-pharmacological interventions for sleep promotion in hospitalized children. Cochrane Database Syst. Rev..

[22] Gomberg J, Raviv A, Fenig E, Meiri N (2020). Saving costs for hospitals through medical clowning: A study of hospital staff perspectives on the impact of the medical clown. Clin. Med. Insights Pediatr..

[23] Lopes-Júnior LC (2020). Effectiveness of hospital clowns for symptom management in paediatrics: Systematic review of randomised and non-randomised controlled trials. BMJ.

[24] Vagnoli L, Caprilli S, Robiglio A, Messeri A (2005). Clown doctors as a treatment for preoperative anxiety in children: A randomized, prospective study. Pediatrics.

[25] Golan G, Tighe P, Dobija N, Perel A, Keidan I (2009). Clowns for the prevention of preoperative anxiety in children: A randomized controlled trial. Paediatr. Anaesth..

[26] Dionigi A, Sangiorgi D, Flangini R (2014). Clown intervention to reduce preoperative anxiety in children and parents: A randomized controlled trial. J. Health Psychol..

[27] Kocherov S (2016). Medical clowns reduce pre-operative anxiety, post-operative pain and medical costs in children undergoing outpatient penile surgery: A randomised controlled trial. J. Paediatr. Child Health.

[28] Wolyniez I (2013). The effect of a medical clown on pain during intravenous access in the pediatric emergency department: A randomized prospective pilot study. Clin. Pediatr. (Phila).

[29] Meiri N, Ankri A, Hamad-Saied M, Konopnicki M, Pillar G (2016). The effect of medical clowning on reducing pain, crying, and anxiety in children aged 2–10 years old undergoing venous blood drawing—A randomized controlled study. Eur. J. Pediatr..

[30] Meiri N (2017). Assistance of medical clowns improves the physical examinations of children aged 2–6 years. Isr. Med. Assoc. J..

[31] Heilbrunn BR (2014). Reducing anxiety in the pediatric emergency department: A comparative trial. J. Emerg. Med..

[32] Vagnoli L, Caprilli S, Messeri A (2010). Parental presence, clowns or sedative premedication to treat preoperative anxiety in children: What could be the most promising option?. Paediatr. Anaesth..

[33] Rennick JE (2018). A pilot randomized controlled trial of an intervention to promote psychological well-being in critically ill children: Soothing through touch, reading, and music. Pediatr. Crit. Care Med..

[34] Sánchez JC (2017). Effects of a humor therapy program on stress levels in pediatric inpatients. Hosp. Pediatr..

[35] Saliba FG (2016). Salivary cortisol levels: The importance of clown doctors to reduce stress. Pediatr. Rep..

[36] Barkmann C, Siem A-K, Wessolowski N, Schulte-Markwort M (2013). Clowning as a supportive measure in paediatrics—A survey of clowns, parents and nursing staff. BMC Pediatr..

[37] Arriaga P, Melo AS, Caires S (2020). The effects of hospital clowning on physical and emotional states of pediatric patients during chemotherapy treatment. Child Youth Care Forum.

[38] Friedler S (2011). The effect of medical clowning on pregnancy rates after in vitro fertilization and embryo transfer. Fertil. Steril..

[39] Jacobs S (2016). Pilot study of massage to improve sleep and fatigue in hospitalized adolescents with cancer. Pediatr. Blood Cancer.

[40] Gottschlich M (2014). The effect of healing touch on sleep patterns of pediatric burn patients: A prospective pilot study. J. Sleep Disord. Treat Care.

[41] White MA, Wear E, Stephenson G (1983). A computer-compatible method for observing falling asleep behavior of hospitalized children. Res. Nurs. Health.

[42] Papaconstantinou EA, Hodnett E, Stremler R (2018). A behavioral-educational intervention to promote pediatric sleep during hospitalization: A pilot randomized controlled trial. Behav. Sleep Med..

[43] Kasem Ali Sliman R, Meiri N, Pillar G (2023). Medical clowning in hospitalized children: A meta-analysis. World J. Pediatr..

[44] Kalmbach DA, Anderson JR, Drake CL (2018). The impact of stress on sleep: Pathogenic sleep reactivity as a vulnerability to insomnia and circadian disorders. J. Sleep Res..

[45] Meiri N (2017). Fear of clowns in hospitalized children: Prospective experience. Eur. J. Pediatr..

[46] Hering E, Pritsker I, Gonchar L, Pillar G (2009). Obesity in children is associated with increased health care use. Clin. Pediatr. (Phila).

[47] Kheirandish-Gozal L (2013). Obstructive sleep apnea in children is associated with severity-dependent deterioration in overnight endothelial function. Sleep Med..

[48] Kruizinga, M. D. *et al.* The impact of lockdown on pediatric ED visits and hospital admissions during the COVID19 pandemic: A multicenter analysis and review of the literature. *Eur. J. Pediatr.* **180**, 2271–2279 (2021). 10.1007/s00431-021-04015-0 PMC7959585 33723971

